# Artificial intelligence in the EU Safe Hearts Plan: prediction alone is not enough

**DOI:** 10.1016/j.lanepe.2026.101784

**Published:** 2026-07-27

**Authors:** Hannah van Kolfschooten, Georges Hattab, Franziska Bächler, Giulio Stefanini

**Affiliations:** a Centre for Life Sciences Law, University of Basel, Peter Merian-Weg 8, 4052, Basel, Switzerland; b Centre for Artificial Intelligence in Public Health Research, Robert Koch Institute, Berlin Germany; c Department of Mathematics and Computer Science, Freie Universität Berlin, Berlin, German; d Department of Biomedical Sciences, Humanitas University, Via Rita Levi Montalcini 4, 20072 Pieve Emanuele, Milan, Italy; e Cardio Center, IRCSS Humanitas Research Hospital, Rozzano, Milan, Italy

**Keywords:** Digital health, Artificial intelligence, EU policy, Cardiovascular disease, Patients' rights

## Abstract

The European Commission's Safe Hearts Plan positions AI as a key tool for enhancing the prevention, diagnosis, and care of cardiovascular disease. Although AI presents significant opportunities, the Safe Hearts Plan may be overstating its impact by prioritising technological capacity over implementation readiness. This Viewpoint examines the role assigned to AI within the Safe Hearts Plan through a policy and legal analysis, drawing on EU policy documents, WHO/Europe reports, and literature on AI governance and cardiovascular care. It argues that a persistent “benefits gap” exists between AI's technical performance and its real-world clinical impact, reflecting institutional, operational, and legal barriers to implementation. The Viewpoint further analyses how legal uncertainty surrounding regulation, liability, and patients' rights may hinder the responsible integration of AI into cardiovascular care. It concludes that AI should be embedded within robust legal, institutional, and operational frameworks to improve cardiovascular outcomes rather than simply generate more accurate predictions.

## Introduction

On 16 December 2025, the European Commission launched the Safe Hearts Plan, the first comprehensive European strategy dedicated to cardiovascular health.^1^ Cardiovascular diseases remain the leading cause of death in the European Union (EU), accounting for around 1.7 million deaths each year.^2^ The strategy aims to strengthen prevention, early detection, and treatment, and encourage Member States to develop national cardiovascular health strategies. Within the Safe Hearts Plan, artificial intelligence (AI) is positioned as an important tool for enhancing prevention, diagnosis, and disease management throughout the cardiovascular care pathway.

While AI has the potential to improve cardiovascular care, its prominence within the strategy risks overstating its likely impact. Cardiovascular outcomes depend not only on predictive tools but also on health system capacity, workforce readiness, legal frameworks, and the broader social determinants of health. WHO/Europe has warned that AI is being introduced into health systems without adequate legal safeguards, while important questions regarding liability and accountability remain unresolved within the EU.^3^ We argue that the Safe Hearts Plan risks overestimating the contribution of AI to cardiovascular health by prioritising predictive capacity over implementation readiness.

This article adopts a narrative policy and legal analysis approach, drawing on EU policy documents, WHO/Europe reports, and literature relating to AI governance and cardiovascular care. First, we consider the functions assigned to AI across the cardiovascular care pathway. Then, we analyse the gap between prediction and improved care. Finally, we examine the legal and regulatory implications for patients’ rights in Europe. Without adequate governance and patient protection, AI could generate predictions that do not improve cardiovascular outcomes.

## AI across the cardiovascular care pathway

AI is an attractive prospect for cardiovascular policy due to the big potential of AI applications throughout the entire care pathway. The Safe Hearts Plan is structured around three pillars, all of which are expected to involve AI: prevention, early detection and screening, and treatment and care.

Within the prevention pillar, AI is positioned as a tool for predicting population-level risk. By analysing large datasets (e.g. electronic health records, lifestyle and environmental data), AI systems can identify individuals at increased cardiovascular risk before symptoms appear, enabling more personalised prevention. This potential is closely linked to the development of interoperable data infrastructures under the European Health Data Space (EHDS).^4^^,^^5^ However, identifying individuals at high-risk of cardiovascular diseases and events only contributes to improved outcomes where health systems can deliver timely and effective preventive interventions.

In the early detection and screening pillar, AI is presented as a tool for analysing clinical and imaging data to identify early signs of disease. Cardiovascular imaging is one of the most advanced applications in this area, supporting the interpretation of echocardiograms, CT scans, and other diagnostic tests.^6^ While these tools may improve diagnostic accuracy or efficiency, their impact depends on how they are integrated into clinical workflows.^7^ Increased detection does not necessarily translate into improved outcomes, especially where early findings are not linked to evidence-based treatment strategies or where health systems lack the capacity to respond effectively.

Within the treatment and care pillar, AI is positioned as an enabler of personalised medicine and more efficient healthcare delivery. AI systems can support clinical decision-making, optimise treatment pathways, and enable remote monitoring via digital health technologies.^8^ However, these applications require stable digital infrastructures, sufficient workforce capacity and training, integration of AI-related competencies into medical education, and effective coordination across care pathways. Without these conditions, AI could complicate clinical practice rather than improving the quality or continuity of care. More broadly, across the cardiovascular care pathway, the role of AI is characterised by a recurring tension between implementation challenges (Panel 1).

Panel 1 **AI in cardiovascular health: benefits, limitations, and implementation challenges across the care pathway.**

**Prevention:**

**Potential benefits**

• Population-level risk prediction using large datasets
• Early identification of individuals at elevated cardiovascular risk
• More targeted and personalised prevention strategies
• Opportunities to leverage interoperable health data infrastructures, including the European Health Data Space (EHDS)

**Limitations and risks**

• Improved risk identification does not necessarily translate into better health outcomes
• Over-reliance on predictive outputs may influence clinical and policy decision making
• Risk of diverting attention from broader population-level public health interventions and social determinants of health

**Implementation challenges**

• Translating risk predictions into effective preventive interventions ('benefits gap')
• Dependence on health system capacity and access to primary care
• Alignment with existing public health priorities and prevention strategies
• Risk of technological solutionism

**Early detection and screening:**

**Potential benefits**

• Improved analysis of clinical, imaging, and diagnostic data
• Increased diagnostic accuracy and efficiency
• Earlier identification of cardiovascular disease and risk factors

**Limitations and risks**

• Detection of disease without clearly established treatment pathways
• Increased demand for follow-up investigations and clinical resources
• Uncertainty regarding the management of early-stage or incidental findings

**Implementation challenges**

• Integration into existing clinical workflows
• Variability in performance across datasets, populations, and health-care settings
• Dataset shift and declining performance over time
• Limited prospective and real-world validation
• Weak evidence linking diagnostic accuracy improvements to meaningful patient outcomes

**Treatment and management:**

**Potential benefits**

• Clinical decision support for diagnosis and treatment selection
• More personalised treatment and disease-management strategies
• Potential improvements in efficiency and resource allocation
• Enhanced remote monitoring and integration with digital health technologies

**Limitations and risks**

• Workflow disruption and increased clinical complexity
• Over-reliance on algorithmic recommendations
• Unequal access to AI-enabled care across health systems and populations

**Implementation challenges**

• Need for robust digital infrastructure and interoperability
• Workforce training and AI literacy requirements
• Coordination across providers and care pathways
• Sustainable funding and reimbursement models
• The ‘last-mile problem’ of translating AI outputs into clinical action
• Institutional, organisational, and governance constraints

## From technical performance to health outcomes: the implementation challenge

Translating predictive capacity into improved care reflects a well-documented “benefits gap” in the use of AI in healthcare. Systems that perform well in controlled or retrospective settings often do not deliver meaningful improvements once deployed in real-world clinical environments.^9^ This gap is not attributable to a single bottleneck, but reflects structural challenges in how AI is evaluated, implemented, and governed in health systems. Recent cardiovascular AI guidance documents, including those of the European Society of Cardiology, similarly emphasise data quality, external validation, implementation context, and evidence of clinical benefit as prerequisites for trustworthy AI integration.^10^^,^^11^

First, the relationship between technical performance and clinical benefit is not well understood. Evaluation frameworks tend to equate predictive accuracy with effectiveness. However, they fail to capture the effects on decision-making, quality of care, and patient outcomes, as also reflected in the several emerging AI-specific evaluation and reporting frameworks.^12^^,^^13^ Furthermore, the evidence base underpinning many AI systems is limited. Demonstrations of high performance are still predominantly based on retrospective validation studies, whereas prospective evaluations in real-world clinical settings are rare and randomised evaluations are even rarer.^14^ AI systems often perform differently across institutions due to variation in patient populations, coding practices, equipment, and workflow design. They are also vulnerable to dataset shift, whereby changes in clinical practice or patient populations reduce performance over time.^15^ In practice, this creates uncertainty about whether AI systems deliver meaningful clinical benefits once integrated into care.

Second, current policy disproportionately emphasises data infrastructure, while underestimating the broader conditions necessary for implementation. This is reflected in what has been termed as the “last mile problem”: many AI tools never progress beyond the development or pilot stage, and even those that do often prove difficult to integrate into routine care.^16^^,^^17^ Initiatives such as the European Health Data Space (EHDS) aim to improve access to, and interoperability of, health data, and are often presented as key enablers of AI innovation.^18^ However, data availability alone is insufficient. The quality and standardisation of health data also remain uneven across Europe, affecting the reliability and representativeness of datasets used to develop AI systems. The effective use of AI requires clinical workflows that can integrate algorithmic outputs, sustainable funding, and a workforce capable of interpreting and acting on AI-supported decisions. Yet, WHO/Europe reports that only a minority of countries provide AI training for health professionals,^19^ while implementation funding also remains limited.^20^ These findings suggest that the main constraints on the use of AI in healthcare are institutional rather than technological. More broadly, AI may require forms of care delivery organised around continuous data generation, rather than simply being added to existing healthcare structures.

Third, the prominence of AI in health policy risks reinforcing a form of technological solutionism. Cardiovascular disease is influenced by social, economic, and environmental factors, and improvements in outcomes depend on various interventions, such as prevention policies, increased primary care capacity, and equitable access to standard of care services. If AI becomes the centrepiece of cardiovascular strategies, there is a risk that technological approaches will overshadow these established public health measures. AI should therefore be understood as operating within—rather than replacing—established cardiovascular prevention frameworks.^21^ At the same time, AI deployment remains concentrated in well-resourced settings, risking the reinforcement of existing inequalities.^22^

These dynamics show that AI's effectiveness depends less on technical performance alone than on health system capacity.

## Legal uncertainty and patients’ rights

These implementation challenges have a distinct legal dimension, as reflected by the ongoing legal uncertainty surrounding responsibility, oversight, liability, informed consent, post-market monitoring, and patient protection in the context of medical AI. According to the WHO/Europe report on AI readiness, legal uncertainty is the most frequently reported barrier to the adoption of AI, having been identified by 86% of responding states.^23^ However, only 8% have developed health-specific liability frameworks for AI.^24^ While existing legal frameworks, including professional liability and EU product liability law, continue to apply, uncertainty remains regarding how these regimes operate in practice for AI systems.^23^ This lack of legal clarity affects not only adoption, but also shapes how AI systems are integrated into clinical practice and whether they can be used safely and responsibly.

Within the EU, this uncertainty is exacerbated by the blurred boundaries surrounding what constitutes “medical AI”. Consumer-facing technologies illustrate this problem. For example, wearables that monitor heart rate, rhythm, or physical activity are increasingly being used to inform health-related decisions; however, their regulatory classification depends on the manufacturer's intended purpose rather than their actual use. Consequently, devices that influence help-seeking behaviour or self-management may fall outside of medical device regulation if they are marketed as lifestyle products.^25^ A similar challenge arises with general-purpose AI systems, such as large language models (LLMs). These tools are widely used to interpret symptoms, explain test results or provide health-related advice, despite not being designed or regulated as medical devices. This creates a gap between legal categorisation and real-world impact, and raises concerns about patient protection.^26^

These concerns are further complicated by the evolving EU regulatory framework governing digital health technologies. The EU AI Act^27^ introduces a risk-based regulatory system that classifies many healthcare AI systems as high risk, subjecting them to stricter requirements relating to safety, transparency, risk management, data governance, and human oversight, and post-market monitoring.^28^ At the same time, questions remain regarding how the AI Act will interact in practice with existing sector-specific frameworks, such as the Medical Devices Regulation (MDR) and the In Vitro Diagnostic Medical Devices Regulation (IVDR), as well as how responsibilities will be allocated across developers, manufacturers, healthcare providers, and deployers in clinical settings. Recent MDCG/AIB guidance clarifies that the AI Act and MDR/IVDR apply complementarily to high-risk medical AI systems, although key standards remain under development.^29^

For patients, these legal uncertainties translate into concrete risks. The growing use of AI in cardiovascular care raises concerns relating to transparency, accountability, equality, patient autonomy and informed consent, and effective redress. Patients may not be aware of when AI is involved in diagnosis or treatment decisions, or of how such systems influence clinical judgement. Where harm occurs, it may be difficult in practice to determine responsibility across clinicians, healthcare providers, or technology developers. Furthermore, if trained on unrepresentative data, AI systems may reproduce or amplify existing health inequalities, particularly given the well-documented differences in cardiovascular risk across demographic and socioeconomic groups. Finally, meaningful informed consent is undermined when patients lack clarity about how decisions affecting their care are made.^29^

These developments reveal a fragmented legal landscape. Member States are being encouraged to integrate AI into cardiovascular strategies, despite continuing uncertainty regarding the interaction of evolving regulatory and liability frameworks. For patients, this raises fundamental questions about access to safe AI and the availability of meaningful protection when these systems malfunction.

## Conditions for the responsible integration of AI into national cardiovascular strategies

The Safe Hearts Plan encourages Member States to develop national cardiovascular strategies in which AI may play an increasing role. However, it provides limited guidance on how AI should be integrated to improve health outcomes. Member States should therefore embed AI within clear legal, institutional, and operational conditions.^30^

First, the adoption of AI should be linked to clearly defined needs within the healthcare system and supported by evidence of clinical benefit. National strategies should stipulate that AI applications demonstrate added value in addressing specific cardiovascular challenges, such as reducing diagnostic delays or improving risk stratification. This should be achieved using endpoints that go beyond predictive accuracy to include clinical outcomes, workflow impact, or cost-effectiveness. Existing health technology assessment (HTA) frameworks should demand evidence of the impact on clinical outcomes and care pathways, rather than focusing solely on predictive performance. This will ensure that technical development remains aligned with regulatory compliance and clinical safety from the outset.

Second, governments should establish integrated governance frameworks that align regulatory oversight with clinical use. These frameworks must go beyond coordination and explicitly allocate legal responsibility to developers, manufacturers, healthcare providers, and public authorities. In practice, this requires clarifying how existing liability regimes, such as those relating to medical devices, professional and product liability, apply to AI-assisted clinical decisions. Where necessary, these regimes should be adapted to address issues such as opacity, continuous learning and distributed decision-making. Health-specific guidance is required on how existing legal frameworks apply in clinical practice, including guidance on liability, informed consent, and professional standards. Furthermore, governance frameworks should incorporate requirements for local validation and continuous performance monitoring to ensure that AI systems are tested on representative populations and are subject to ongoing auditing for performance drift, subgroup disparities, and unintended effects. These obligations should be embedded in post-market surveillance and linked to clearly defined responsibilities for stakeholders.

Third, investments in health data infrastructure, particularly under the EHDS, should be accompanied by implementation capacity requirements and robust data governance. Member States should ensure that AI deployment is supported by interoperable electronic health records, integration into clinical workflows, and sustainable reimbursement models. At the same time, clear legal rules are needed regarding data access, secondary use, and accountability for data quality, including responsibilities for bias mitigation and data stewardship. Without alignment between data governance, clinical practice, and financing, AI tools are unlikely to move beyond pilot stages.

Fourth, workforce readiness should be addressed through targeted training and professional guidance. National strategies should include competencies for clinicians using AI systems, covering the interpretation of outputs, awareness of limitations, and the management of uncertainty. Building on emerging initiatives to integrate AI literacy into medical education and continuing professional development, is essential to mitigate risks such as automation bias or overreliance on algorithmic outputs. Workforce training must address alignment between human-AI interaction and go beyond basic literacy. This involves designing interfaces that preserve human agency and enable clinicians to understand the ‘causality’ behind AI-driven cardiovascular risks.^31^

Finally, national strategies must operationalise patients’ rights in the context of AI while ensuring equitable access and outcomes. This involves translating principles such as transparency, accountability, and autonomy into enforceable obligations within clinical practice. Patients should be informed when AI systems meaningfully contribute to diagnosis, risk prediction, or treatment decisions, and they should be given access to understandable information about their role and the limitations of these systems. Member States should also establish clear accountability pathways for when harm occurs, including accessible redress mechanisms. Equity should also be treated as a measurable objective of AI deployment. Member States should ensure that AI systems are evaluated across relevant demographic and socioeconomic groups, identifying and addressing any disparities in performance or access. As AI deployment remains concentrated in well-resourced settings, targeted measures may be required to prevent the reinforcement of existing inequalities in cardiovascular health.

## Conclusion

The Safe Hearts Plan reflects a broader shift in European health policy, positioning AI as a key tool for improving care. However, the effectiveness of AI in cardiovascular health must be considered within the context of the systems into which it is introduced. Technical performance does not automatically result in better health outcomes, and it can create new forms of complexity, inequality and risk if it is not supported by adequate legal, institutional and operational foundations. The central challenge is therefore not whether AI can improve cardiovascular care, but under which legal, institutional, and operational conditions it can do so. Without addressing the benefits gap, Europe risks generating more accurate predictions without achieving better cardiovascular outcomes.

## Contributors

HvK conceptualized the research, conducted the formal analysis, and wrote the original draft. HvK, GH, FB, and GS critically revised it for important intellectual content and contributed to the final writing and editing. HvK, GH, FB, and GS were responsible for the decision to submit the manuscript.

## Declaration of interests

HvK declares membership of the WHO Technical Advisory Group on AI for Health (unpaid). WHO had no role in the writing of the manuscript. GH, FB, and GS declare no competing interests.

## Appendix A Supplementary data

Dutch Translate Abstract

German Translate Abstract

Italian Translate Abstract
