# Supplementary material for: Artificial intelligence in the EU Safe Hearts Plan: prediction alone is not enough
Source: Lancet Reg Health Eur. 2026 Jul 27;67:101784. doi: 10.1016/j.lanepe.2026.101784 (PMC13446344; doi:10.1016/j.lanepe.2026.101784)
Supplement: Supplementary file 1 [file mmc1.docx]

This translation in Italian was submitted by the authors and we reproduce it as supplied. It has not been peer reviewed. Our editorial processes have only been applied to the original abstract in English, which should serve as reference for this manuscript.

**Italiano:**

Il Piano Safe Hearts della Commissione Europea presenta l’IA come uno strumento fondamentale per migliorare la prevenzione, la diagnosi e il trattamento delle malattie cardiovascolari. Sebbene l’IA offra opportunità promettenti per il percorso di cura cardiovascolare, il Piano rischia di sopravvalutarne l’impatto, ponendo maggiore enfasi sulle capacità tecnologiche rispetto alla concreta implementazione nella pratica sanitaria. Vi sono indicazioni di un persistente “benefits gap”: anche sistemi di IA altamente performanti non si traducono automaticamente in migliori esiti clinici nella pratica, a causa di ostacoli istituzionali, operativi e giuridici. Le strategie nazionali in materia cardiovascolare non dovrebbero quindi considerare l’IA come una soluzione autonoma, ma integrarla all’interno di chiari quadri giuridici, organizzativi e operativi che ne consentano un utilizzo efficace e responsabile. Ciò richiede, tra le altre cose, di collegare l’adozione dell’IA a benefici clinici dimostrabili, definire in termini chiari le responsabilità, garantire validazione locale e monitoraggio continuo, investire nelle competenze del personale e nelle infrastrutture dei dati, nonché assicurare una concreta tutela dei diritti dei pazienti e dell’equità. In assenza di queste condizioni, l’IA rischia di generare più dati e previsioni, senza migliorare gli esiti cardiovascolari.
