# Supplementary material for: Artificial intelligence in the EU Safe Hearts Plan: prediction alone is not enough
Source: Lancet Reg Health Eur. 2026 Jul 27;67:101784. doi: 10.1016/j.lanepe.2026.101784 (PMC13446344; doi:10.1016/j.lanepe.2026.101784)
Supplement: Supplementary file 2 [file mmc2.docx]

This translation in German was submitted by the authors and we reproduce it as supplied. It has not been peer reviewed. Our editorial processes have only been applied to the original abstract in English, which should serve as reference for this manuscript.

**Deutsch:**

Der “Safe Hearts Plan” der Europäischen Kommission stellt die KI als wichtiges Instrument für die Verbesserung der Prävention, Diagnose und Behandlung von Herz-Kreislauf-Erkrankungen dar. Obwohl KI vielversprechende Möglichkeiten im Bereich der Behandlung bietet, besteht die Gefahr, dass der Plan ihren tatsächlichen Einfluss überschätzt, indem er einerseits die technologische Leistungsfähigkeit stärker hervorhebt und andererseits die Bereitschaft der praktischen Umsetzung im Gesundheitswesen vernachlässigt. Es gibt Beweise über die Existenz einer anhaltende „Benefits Gap“(Leistungslücke), anhand welcher festgestellt werden kann, dass die Nutzung leistungsstarker KI-Systeme in der Praxis häufig nicht zu besseren klinischen Ergebnissen führt. Das Problem ist auf die institutionellen, operativen und rechtlichen Hindernisse zurückzuführen. Nationale Strategien zur Bekämpfung von Herz-Kreislauf-Erkrankungen sollten deswegen KI nicht als eigenständige Lösung betrachten, sondern jene in klare rechtliche, organisatorische und operative Rahmenbedingungen eingliedern, wodurch ein verantwortungsvoller und wirksamer Einsatz der Technologie ermöglicht werden kann. Diese Eingliederung beinhaltet die Verwendung der KI mit dem nachweisbaren klinischen Nutzen zu verbinden, klare Verantwortlichkeiten festzulegen, lokale Nachweise und kontinuierliches Monitoring zu gewährleisten und in Personalressourcen, Dateninfrastrukturen sowie die konkrete Durchsetzung der Gleichbehandlung von Patienten und deren Rechte zu investieren. Ohne Erfüllung dieser Voraussetzungen ist zu befürchten, dass die KI Daten und Vorhersagen erzeugen wird, ohne gleichzeitig die kardiovaskulären Gesundheitsergebnisse tatsächlich zu verbessern.
