# Supplementary material for: Artificial intelligence in the EU Safe Hearts Plan: prediction alone is not enough
Source: Lancet Reg Health Eur. 2026 Jul 27;67:101784. doi: 10.1016/j.lanepe.2026.101784 (PMC13446344; doi:10.1016/j.lanepe.2026.101784)
Supplement: Supplementary file 3 [file mmc3.docx]

This translation in Dutch was submitted by the authors and we reproduce it as supplied. It has not been peer reviewed. Our editorial processes have only been applied to the original abstract in English, which should serve as reference for this manuscript.

**Nederlands:**

Het “Safe Hearts Plan” (“Plan voor een veilig hart”) van de Europese Commissie positioneert AI als een belangrijk instrument voor de preventie, diagnose en behandeling van hart- en vaatziekten. Hoewel AI veelbelovende mogelijkheden biedt binnen het cardiovasculaire zorgtraject, dreigt het plan de impact ervan te overschatten door technologische capaciteit centraal te stellen boven daadwerkelijke implementatie in de zorgpraktijk. Er bestaan aanwijzingen voor een aanhoudende “benefits gap”: goed presterende AI-systemen leiden in de praktijk niet automatisch tot betere klinische uitkomsten, onder meer door institutionele, operationele en juridische belemmeringen. Nationale cardiovasculaire strategieën zouden AI daarom niet moeten benaderen als een op zichzelf staande oplossing, maar moeten inbedden in duidelijke juridische, organisatorische en operationele kaders die verantwoord en effectief gebruik mogelijk maken. Dit vereist onder meer een koppeling tussen AI-adoptie en aantoonbaar klinisch voordeel, lokale validatie en continue monitoring, investeringen in personeelscapaciteit en data-infrastructuur, en een concrete verankering van patiëntenrechten in de wet. Zonder deze randvoorwaarden dreigt AI vooral méér data en voorspellingen te genereren, zonder daadwerkelijk de cardiovasculaire gezondheidsuitkomsten te verbeteren.
